# Beyond the desert sands: decoding the relationship between camels, gut microbiota, and antibiotic resistance through metagenomics

**DOI:** 10.1016/j.soh.2024.100071

**Published:** 2024-07-01

**Authors:** Yan Gao, Jiangchao Wu, Jun Zeng, Xiangdong Huo, Kai Lou

**Affiliations:** a Key Laboratory of Biological Resources and Ecology of Pamirs Plateau, Kashgar 844000, Xinjiang Uygur Autonomous Region, China; b Institute of Applied Microbiology, Xinjiang Academy of Agricultural Sciences, Urumqi 830091, Xinjiang Uygur Autonomous Region, China; c The College of Life and Geographic Sciences, Kashgar University, Kashgar 844000, Xinjiang Uygur Autonomous Region, China; d Xinjiang Laboratory of Special Environmental Microbiology, Urumqi 830091, Xinjiang Uygur Autonomous Region, China

**Keywords:** Metagenomics, Camels, Gut microbiota, Antibiotic resistance

## Abstract

**Background:**

Camels, known as the enduring “ships of the desert,” host a complex gut microbiota that plays a crucial role in their survival in extreme environments. However, amidst the fascinating discoveries about the camel gut microbiota, concerns about antibiotic resistance have emerged as a significant global challenge affecting both human and animal populations. Indeed, the continued use of antibiotics in veterinary medicine has led to the widespread emergence of antibiotic-resistant bacteria, which has worsened through gene transfer.

**Methods:**

This study offers a deeper examination of this pressing issue by harnessing the potent tools of metagenomics to explore the intricate interplay between the camel (*Camelus ferus*) gut microbiota and antibiotic resistance.

**Results:**

Samples from wild camels yielded varying amounts of raw and clean data, generating scaftigs and open reading frames. The camel fecal microbiome was dominated by bacteria (mainly *Bacillota* and *Bacteriodota*), followed by viruses, archaea, and eukaryota. The most abundant genera were the *Bacteroides*, *Ruminococcus*, and *Clostridium*. Functional annotation revealed enriched pathways in metabolism, genetic information processing, and cellular processes, with key pathways involving carbohydrate transport and metabolism, replication, and amino acid transport. CAZy database analysis showed high abundances of glycoside hydrolases and glycosyl transferases. Antibiotic resistance gene (ARG) analysis identified *Bacillota* and *Bacteroidota* as the main reservoirs, with vancomycin resistance genes being the most prevalent. This study identified three major resistance mechanisms: antibiotic target alteration, antibiotic target protection, and antibiotic efflux.

**Conclusion:**

These findings contribute to a broader understanding of antibiotic resistance within animal microbiomes and provide a foundation for further investigations of strategies to manage and mitigate antibiotic resistance.

## Highlights

• Bacillota and Bacteriodota are the most abundant bacterial phyla for immune homeostasis, carbohydrate metabolism, and SCFA production in camels.
• The study identifies antibiotic resistance genes (ARGs) to be predominantly in the bacterial phyla Bacillota and Bacteroidota.
• Vancomycin resistance is of concern, highlighting potential risks from improper antibiotic use in livestock.
• Fecal shedding of the antibiotic-resistant bacteria and ARGs into the environment.
• Antibiotic spread can potentially impact human health through horizontal gene transfer.

## 1 Introduction

Camels, often symbolized as the “ships of the desert,” are a testament to survival in extremis. With an inherent capacity to thrive in some of the most inhospitable terrains on Earth, these unique mammals have been a focal point of scientific interest for centuries. Their ability to cope with prolonged periods of food and water scarcity is facilitated by physiological adaptations such as fat storage in humps, low metabolic rates, and efficient water conservation mechanisms [1]. Yet, a critical component of this survival strategy, which has remained relatively concealed, is the community of microorganisms residing within the camel's gut, known as the gut microbiota. The gut microbiota, a complex ecosystem of bacteria, fungi, viruses, and other microbes, plays a pivotal role in maintaining the overall health of the host and interacts dynamically with the host's physiology, influencing various physiological processes, immune responses, and metabolism [2].

For camels, this internal microcosmos has evolved to suit their specific dietary needs and environmental challenges, aiding in the digestion of roughage, boosting their immune response, and even assisting in temperature regulation [3]. While our knowledge of the impact of gut microbiota on the physiology of camels is still in its infancy, advancements in the field of metagenomics have paved the way for more profound insights [4].

Metagenomics, a revolutionary approach for studying the collective genomes of microbial communities, has extended the scope of microbial ecology research. It allows scientists to decode the genetic material recovered directly from environmental samples, bypassing the need for individual culture of organisms. This ability has been transformative, illuminating the immense diversity and intricate dynamics within the microbial communities of the camel gut [5,6]. Furthermore, the knowledge gained from metagenomic studies of camel gut microbiota has potential implications for advancing our understanding of camel health and enhancing camel welfare in various ways. For instance, probiotic interventions tailored to specific needs of camels can be explored by identifying beneficial microbial strains present in their gut. Probiotics are live microorganisms that, when administered in adequate amounts, confer health benefits to the host by modulating the gut microbiota and improving the overall gut microbial balance [7]. The discovery of probiotic candidates within the camel gut microbiota could lead to innovative strategies to optimize the digestive efficiency of camels, enhance nutrient utilization, and alleviate the impacts of stressors in their environment [8].

However, amidst the fascinating discoveries about the camel gut microbiota, concerns about antibiotic resistance have emerged as a serious global challenge affecting both human and animal populations. Overuse and misuse of antibiotics in human and veterinary medicine have led to the widespread emergence of antibiotic-resistant bacteria, rendering many antibiotics less effective for treating infectious diseases [9] and are widely recognized as a serious global health crisis [10]. The gut microbiota, due to its diversity and high genetic exchange rates, is a potential hotbed for the evolution and dissemination of antibiotic resistance genes (ARGs) [11].

To combat antibiotic resistance effectively, it is crucial to understand the dynamics of ARGs within the camel gut microbiota. Metagenomics has enabled the exploration of ARGs, providing insights into their prevalence and potential transfer between microbes [12]. This knowledge is critical for implementing appropriate measures to preserve the effectiveness of antibiotics in both veterinary and human medicine.

In this context, this study presents an overview of the importance of metagenomics in elucidating the composition and dynamics of camel gut microbiota, with a focus on probiotic potential and antibiotic resistance. An understanding of this interaction is paramount for developing sustainable strategies to manage antibiotic resistance, and thereby, secure the health of our camels and, potentially, our own [13].

## 2 Materials and methods

### 2.1 Animals and sample collection

Fecal samples (LTS01, LTS02, and LTS03) of three wild camels, *Camelus ferus* (one male and two females, body size estimated at 600 kg) were collected in April 2023, in Xinjiang Uygur Autonomous Region, China. Fecal samples were collected from fresh stools. After collection, the samples were promptly placed into 2 mL sterilized centrifuge tubes and transported on dry ice to the laboratory. To ensure their preservation, they were stored at a frigid temperature of −80 ℃, ready for further processing and analysis.

Specifically, this study involved the collection of fresh stool samples from wild camels, identified through their free-roaming behavior in non-domesticated habitats, far from human settlements. The researchers determined that the camels were wild by observing their lack of ear tags, collars, or any other forms of human-made identifiers. Additionally, these camels exhibited wary and unapproachable behavior, typical of non-domesticated animals. Researchers cautiously approached the camels to avoid disturbing them, and collected stool samples immediately after defecation to ensure freshness and avoid contamination. The team documented the sex and approximate body size of each camel from a distance, using binoculars for accurate observation. Sex was determined by the presence or absence of visible genitalia during certain behaviors. Body size was estimated based on shoulder height and body length.

### 2.2 Extraction of DNA and sequencing

DNA extraction from camel fecal samples was performed using the E.Z.N.A.® Stool DNA Kit (D4015, Omega, Inc., Norcross, GA, USA) as per the manufacturer's guidelines. The resulting DNA was purified and stored at −80 ℃. Subsequently, the DNA was subjected to library preparation, including fragmentation, end repair, A-tailing, adapter ligation, and PCR amplification. Quality checks were conducted at various stages, ensuring high-quality libraries. The libraries meeting the criteria were pooled and sequenced on an Illumina PE150 platform to generate the desired data volume.

Specifically, the primers used to detect *tetA* gene (tetracycline resistance) were forward primer (5′-GGTTCACTCGAACGACGTCA-3′) and reverse primer ( 5′-CTGTCCGACAAGTTGCATGA-3′) [14]. The PCR amplification process was undertaken by preparing a master mix that included the DNA template extracted from the camel feces, the specific forward and reverse primers (at a concentration of 10 μmol/L each), dNTPs (200 μmol/L each), MgCl_2_ (1.5 mmol/L or as required by the DNA polymerase), the appropriate buffer, Taq DNA polymerase (1–2.5 U), and nuclease-free water to reach the desired final volume. The thermal cycling conditions used were set up with an initial denaturation at 95 ℃ for 5 min to ensure the DNA strands are separated, followed by 30–35 cycles of denaturation at 95 ℃ for 30 s, annealing at 55–60 ℃ (adjusted based on the primer melting temperature) for 30 s, and extension at 72 ℃ for 1 min. A final extension step at 72 ℃ for 5–10 min ensures that any remaining single-stranded DNA was fully extended. The reaction was then maintained at 4 °C. After amplification, the PCR products were analyzed through gel electrophoresis. A 1%–2% agarose gel was prepared with a suitable DNA stain (e.g. ethidium bromide or SYBR Safe). The PCR products, along with a DNA ladder for size comparison, were loaded into the wells of the agarose gel. Electrophoresis was conducted at 100–120 V until the dye front had migrated an appropriate distance. The gel was then visualized under a UV transilluminator or blue light to detect the presence of bands corresponding to the expected sizes of the resistance genes. Both positive and negative controls were included. The positive control contained DNA with known resistance genes to confirm the PCR had functioned correctly, while the negative control contained no DNA template to check for any contamination. The presence of bands at the expected sizes in the sample lanes, as compared to the positive control and DNA ladder, indicated the presence of specific ARGs in the camel feces [15, 16].

### 2.3 Bioinformatics analysis and statistical methods

To generate high-quality “clean data” for further analysis, the raw sequencing data was pre-processed using Readfq (V8, https://github.com/cjfields/readfq). This process involved the removal of sequence reads with a substantial proportion of low-quality bases, discarding reads containing a certain percentage of “N” bases (representing unknown or undetermined bases), and eliminating reads that significantly overlapped with adapter sequences. Following pre-processing, the *MEGAHIT* software (v1.0.4-beta) was used for assembly analysis. After this, the gene prediction and abundance analysis were performed by *MetaGeneMark* (V3.05) with default parameters [17]. Subsequently, any predicted information with a length < 100 nucleotides was removed from the dataset [18]. Then processing of open reading frame predictions was done using *CD-HIT* software (V4.5.8) to eliminate redundant data and create a non-redundant initial gene catalog. To evaluate the gene expression profile in each sample, the clean data of each sample was aligned to this initial gene catalog using *Bowtie2* (version 2.2.4) with default parameter settings (--end-to-end, --sensitive, –I 200, -X 400) [17]. To finalize the gene catalog (unigenes) for subsequent analysis, genes with reads that were > 2 in each sample were excluded. The abundance of each gene in each sample was determined based on the number of aligned reads and the length of the gene.

### 2.4 Species annotation

Using *DIAMOND* software (version 0.9.9.110) [19], the unigenes sequences were aligned with that of bacteria, fungi, archaea, and viruses from NCBI's NR database (version 2018-01-02) using the blastp parameter with an *e*-value threshold of 1×10^−5^ [20]. The lowest common ancestor algorithm from the *MEGAN* software [21] was applied to resolve multiple alignments and determine the species annotation information. The abundance of each sample at various taxonomic levels (i.e. kingdom, phylum, class, order, family, genus, and species) was determined by combining the lowest common ancestor annotation results with a gene abundance table. The abundance of a specific species in a sample was calculated as the sum of the abundances of all genes annotated as that species, while the number of genes belonging to a specific species in a sample was calculated as the count of genes with non-zero abundance and annotated as that species [22]. We utilized Krona analysis [23], a relative abundance overview, and abundance clustering heatmap for further analysis.

### 2.5 Assessment of functional role of microbiome and ARGs analysis

The unigene sequences were primarily synchronized with diverse functional repositories, including the KEGG database (Version 2018-01-01) [24], the EggNOG database (Version 4.5) [25], and the CAZy database (Version 201801) [26]. The ensuing alignment outcomes were used to identify the topmost BLAST hits [27] for individual sequences, forming the backbone for further exploratory analysis. Utilizing these alignment outcomes, the relative abundance of different functional tiers were computed. The integration of functional annotation results with the gene abundance tabulation resulted in a comprehensive gene number breakdown at each taxonomic level. The enumeration of genes exhibiting a specific function in a given sample was accomplished by identifying non-zero abundance genes within the designated functional annotation. The detailed understanding of functional makeup was accomplished through performing annotated gene statistics, devising a relative abundance overview, and formulating an abundance clustering heatmap. Further, the unigenes sequences were juxtaposed with the Comprehensive Antibiotic Resistance Database (CARD) [28] using the resistance gene identifier [29] software, operating under the default parameters specified by the CARD database. The relative abundance of each antibiotic resistance ontology was determined based on the alignment outcomes and abundance information of the unigenes. To delve deeper into resistance gene traits, an abundance histogram, an abundance clustering heatmap, and an abundance distribution circle map were generated.

## 3 Results

### 3.1 Sequencing information

The samples collected from the camels yielded varying quantities of raw and clean base data (Table S1). Assembly of the cleaned sequences resulted in the generation of scaftigs (Table S2). The findings from the assembly and scaftig length distribution analyses are visually presented in Fig. S1. Each samples had a different number of open reading frames (Fig. S2) and chemistry, some with more start codons and some with more stop codons (Table S3).

### 3.2 Microbial ecology of camel fecal microbiome

**Fig. 1:**
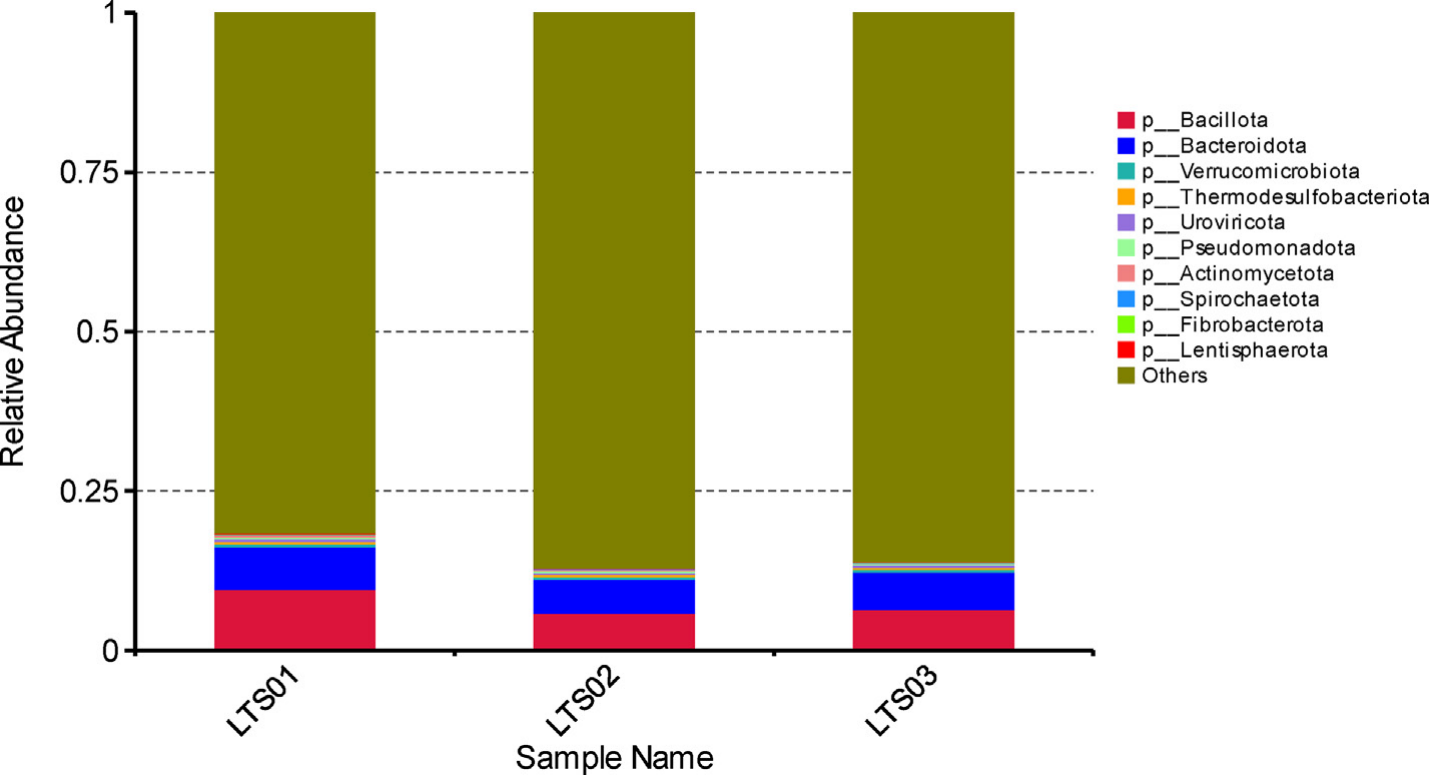
Relative abundance of fecal microbiota at the phylum level in three fecal samples of camels.

**Fig. 2:**
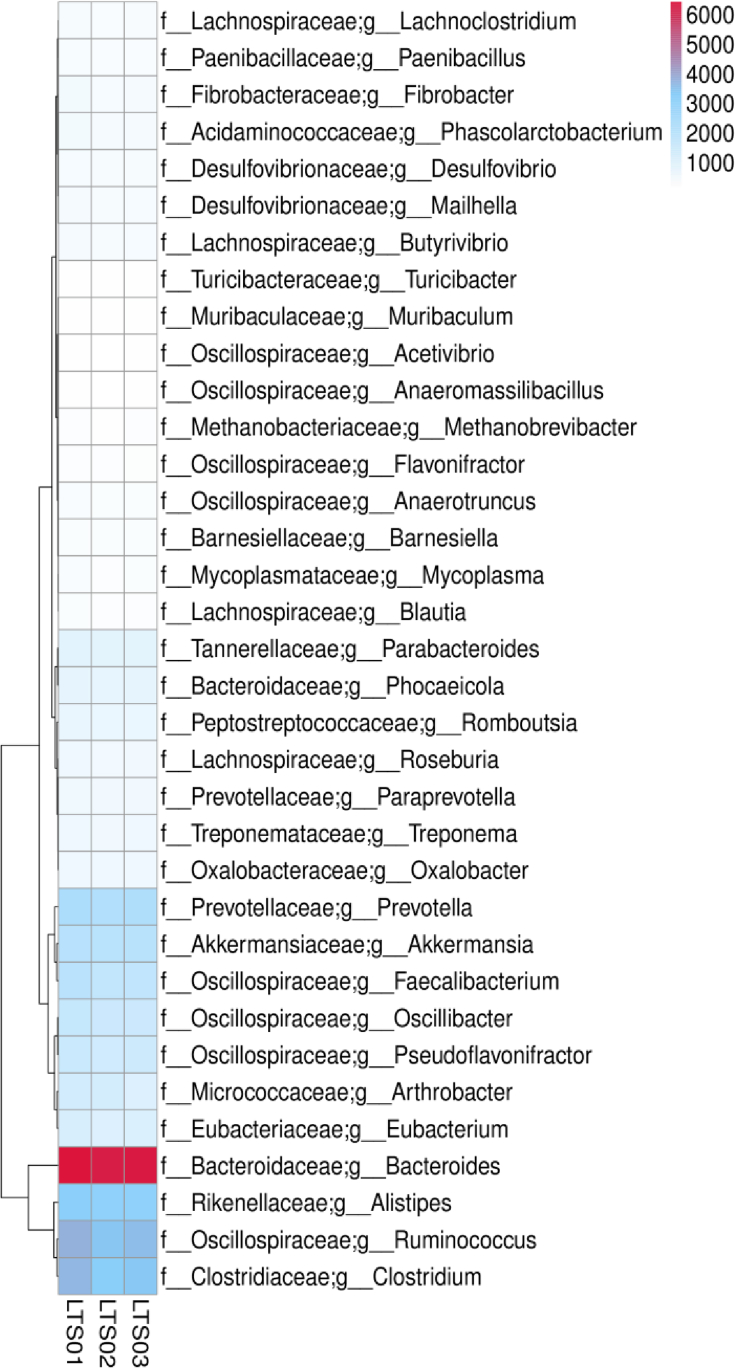
Heatmap of high abundance fecal microbiota at genus level in three fecal samples of camels.

We employed Krona to visualize the species annotation results and effectively present the relative abundance of species at various taxonomic levels in each sample. Our results indicated that camel fecal microbial communities were dominated by bacteria (LTS01 = 20%, LTS02 = 14%, LTS03 = 15%), followed by viruses (LTS01 = 0.5%, LTS02 = 0.4%, LTS03 = 0.4%), archaea (LTS01 = 0.05%, LTS02 = 0.04%, LTS03 = 0.04%) and eukaryota (LTS01 = 0.005%, LTS02 = 0.004%, LTS03 = 0.003%) (Figs. S3 and S4). The most abundant phyla were *Bacillota* and *Bacteriodota* (Fig. 1). Other less abundant phyla were *Verrucomicrobiotap*, *Thermodesulfobacteriotap*, *Uroviricotap*, *Pseudomonadotap*, *Actinomycetotap*, *Spirochaetotap*, *Fibrobacterotap*, and *Lentisphaerota*. At the genus level, there are 11 most abundant genera including *Bacteroides*, *Ruminococcus*, *Clostridium*, *Alistipes*, *Prevotella*, *Akkermansia*, *Faecalibacterium*, *Oscillibacter*, *Pseudoflavonifractor*, *Arthrobacter*, and *Eubacterium* (Fig. 2). The most abundant class, order, family, genus and species were *Clostridia*, *Eubacterialeso*, *Bacteroidaceaef*, *Bacteroidaceae*, and *Clostridiales*, respectively (Fig. S5).

### 3.3 Exploring the functional role of the microbiome in camels

**Fig. 3:**
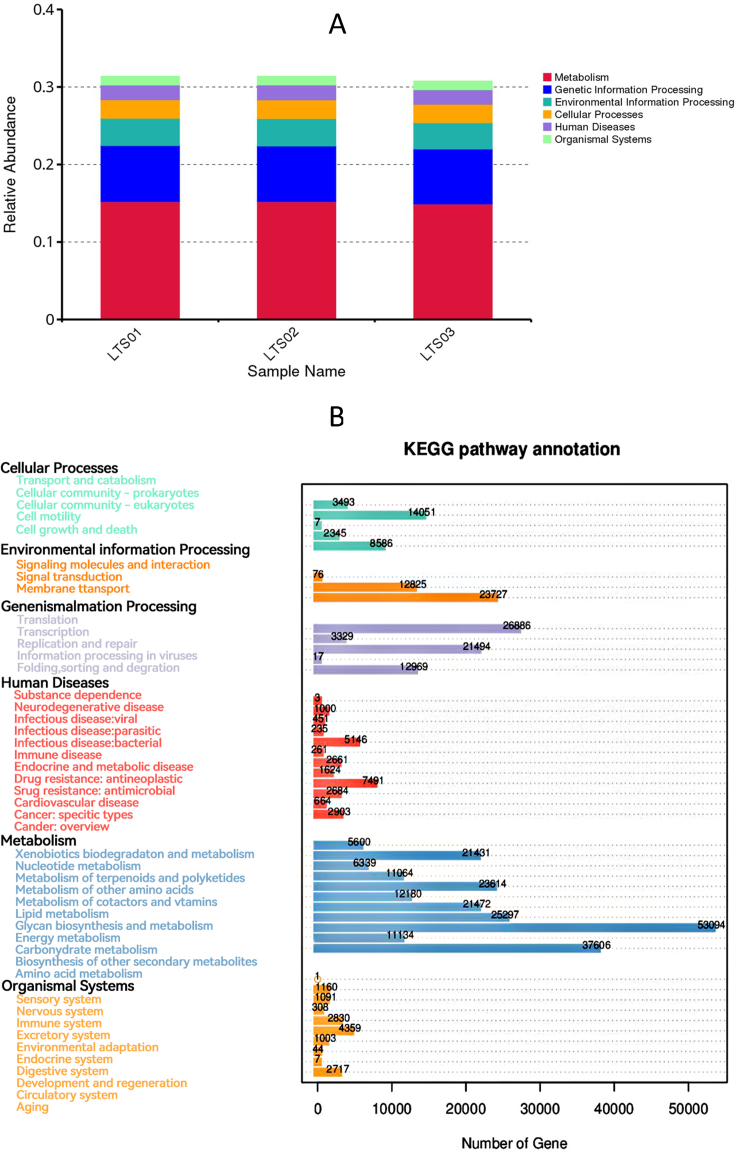
KEGG analysis for three fecal samples of camels. (A) relative abundance of KEGG unique gene level 1. (B) Annotated gene number by KEGG pathway annotation. KEGG: Kyoto Encyclopedia of Genes and Genomes.

**Fig. 4:**
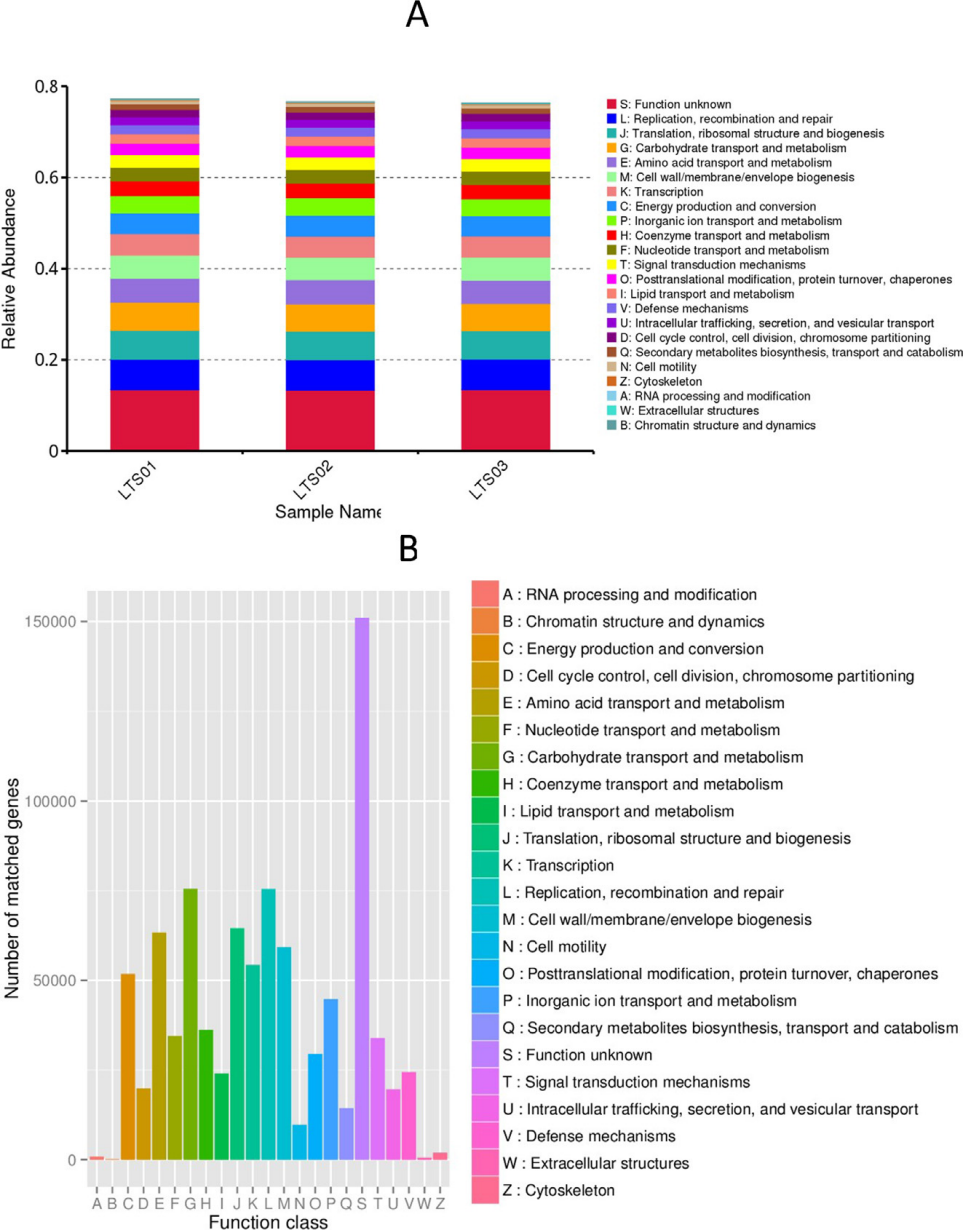
EggNOG analysis for three fecal samples of camels. (A) relative abundance of eggNOGG unique gene level. (B) Number of matched genes for different functions.

**Fig. 5:**
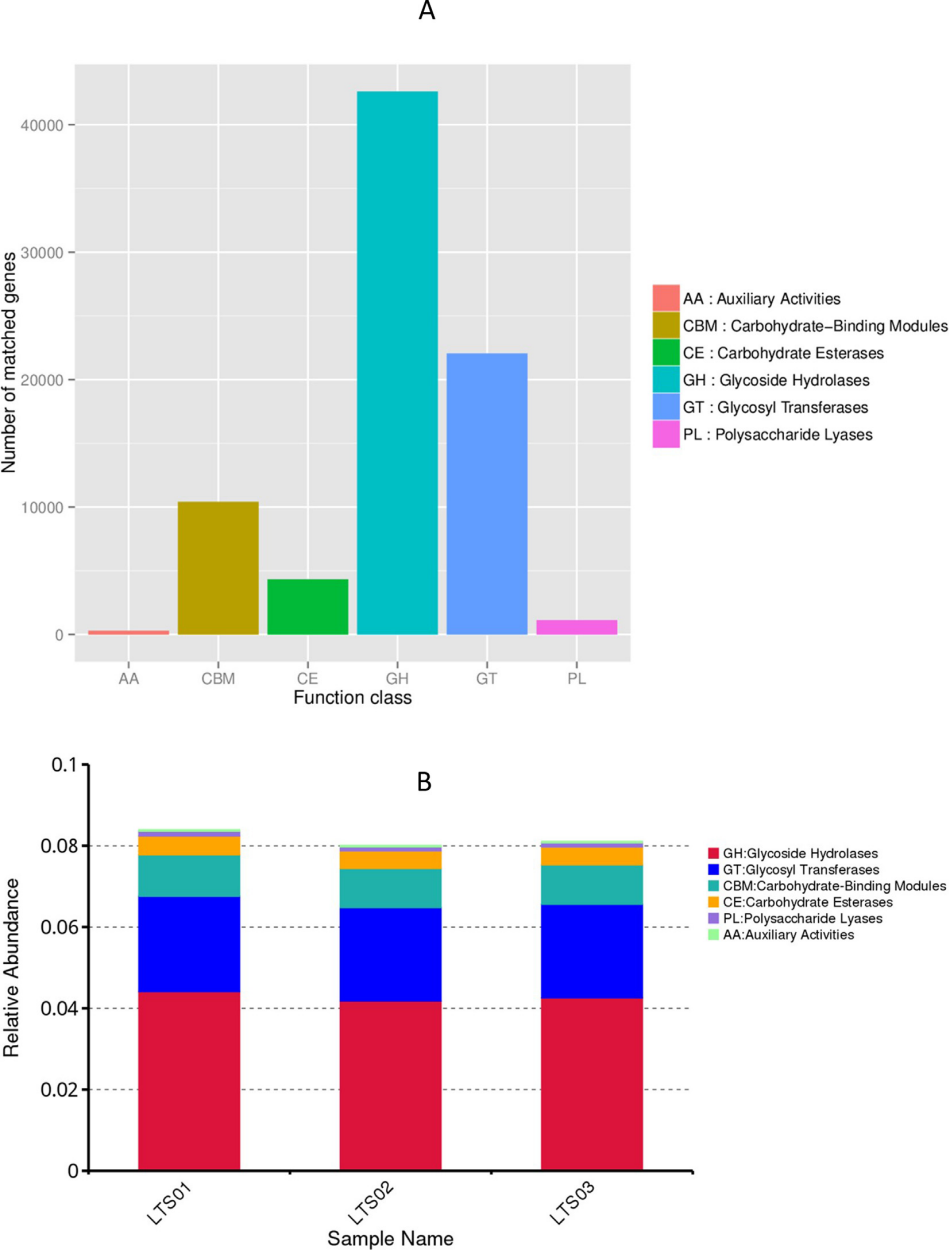
CAZy analysis for three fecal samples of camels. (A) Number of matched genes for different functions. (B) Relative abundance of CAZy unique gene level.

Functional annotations of the identified unigenes was performed using the KEGG, EggNOG, and CAZy databases. The most enriched pathways were concentrated in metabolism related pathways (239,831), followed by genetic information processing related pathways (64,695), environmental information processing related pathways (36,628), cellular processes related pathways (28,482), human diseases related pathways (25,179), and organismal system related pathways (13,521) (Fig. 3). The main pathways with the highest enrichment of basic metabolic function genes were carbohydrate transport and metabolism (75,528), replication, recombination, and repair (75,477), amino acid transport and metabolism (63,339), translation, ribosomal structure and biogenesis (64,514), inorganic ion transport and metabolism (44,782), energy production and conversion (51,765), transcription (54,272), nucleotide transport and metabolism (34,480), posttranslational modification, protein turnover, chaperones (29,459), lipid transport and metabolism (24,023), defense mechanisms (24,424), cell wall/membrane/envelope biogenesis (59,204), signal transduction mechanisms (33,880), coenzyme transport and metabolism (36,183), intracellular trafficking, secretion, and vesicular transport (19,578), cell cycle control, cell division, chromosome partitioning (19,871), secondary metabolites biosynthesis, transport, and catabolism (14,338), cell motility (9750), extracellular structures (590), RNA processing and modification (851), chromatin structure and dynamics (152), and cytoskeleton (190) (Fig. 4). Moreover, the CAZy database analysis revealed the highest abundances of glycoside hydrolases (42,609), glycosyl transferases (22,050), carbohydrate-binding modules (10,404), carbohydrate esterases (4335), polysaccharide lyases (1121), and auxiliary activities (292) (Fig. 5).

### 3.4 Assessment of ARGs analysis

**Fig. 6:**
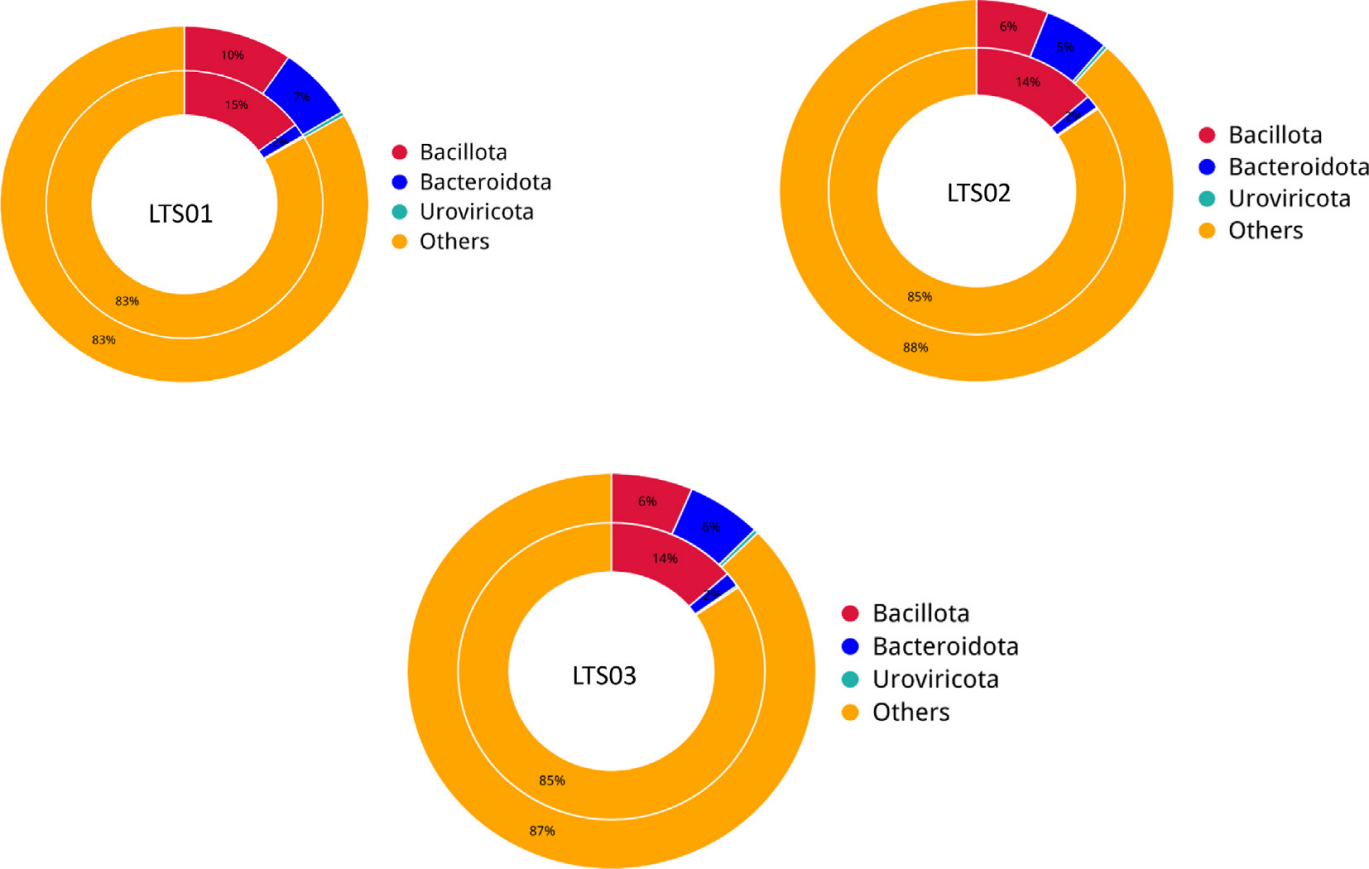
Comparison of the distribution of the ARGs and the bacterial gene sets at the phylum level. The inner circle is the species distribution of ARGs, while the outer circle is the species distribution of all sample genes in the group. Abbreviation: ARGs, antibiotic resistance genes.

**Fig. 7:**
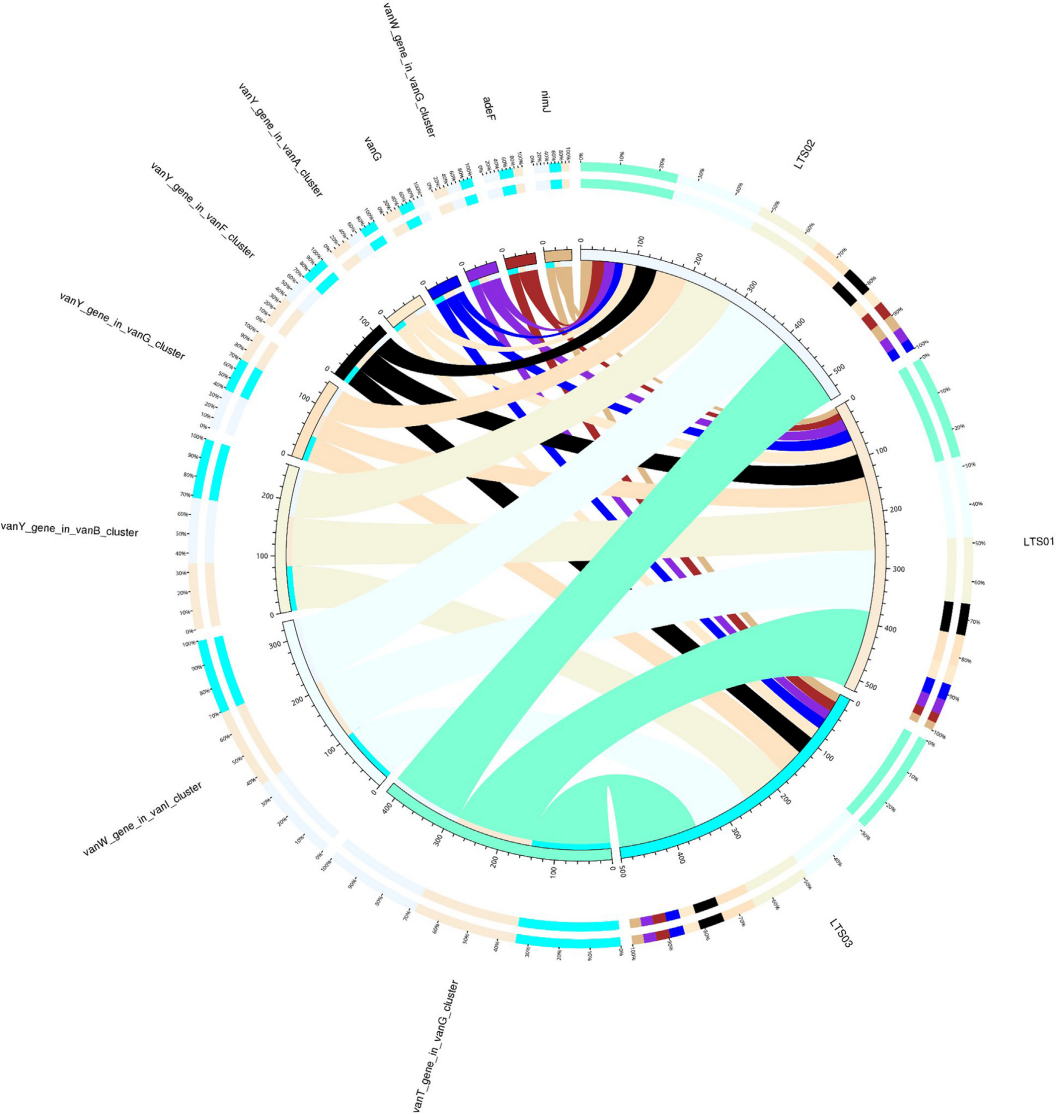
The circular diagram is partitioned into two distinct sections: the right portion displays sample information, while the left section exhibits information related to ARGs. Within the inner circle, diverse colors are utilized to represent distinct samples and ARGs. The scale employed corresponds to the relative abundance of ARGs in parts per million (ppm). On the left-hand side, the sum of the relative abundances of a particular ARG in each sample is depicted, while the right-hand side showcases the cumulative relative abundances of each ARGs within a specific sample. Moving to the outer circle, the left side illustrates the relative percentage content of each sample concerning a particular ARG, while the right side demonstrates the relative percentage content of each ARGs within a specific sample. Abbreviation: ARGs, antibiotic resistance genes.

**Fig. 8:**
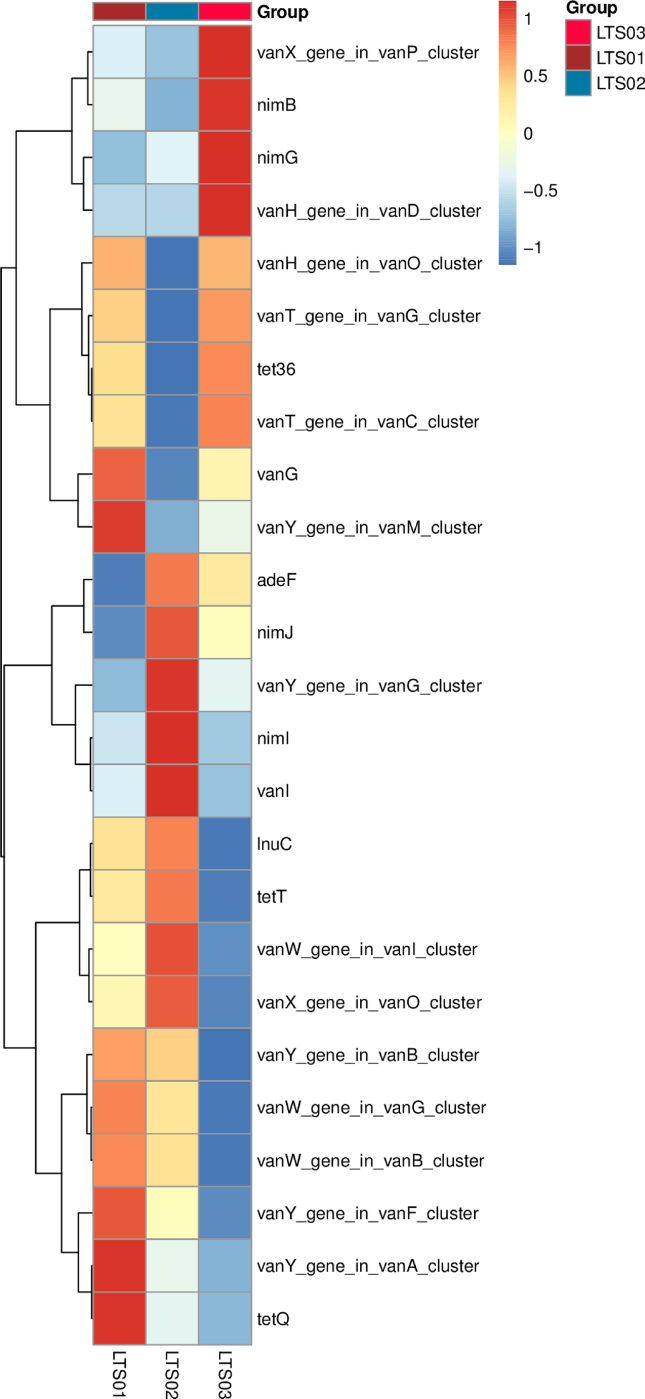
The abundance clustering heatmap of the top 25 ARGs for three fecal samples of the camels. The right vertical axis is the ARG name, the left vertical axis is the ARG clustering tree, and the corresponding value of the intermediate heatmap is the *Z* value of ARG relative abundance in each row after standardized processing. Abbreviation: ARGs, antibiotic resistance genes.

**Fig. 9:**
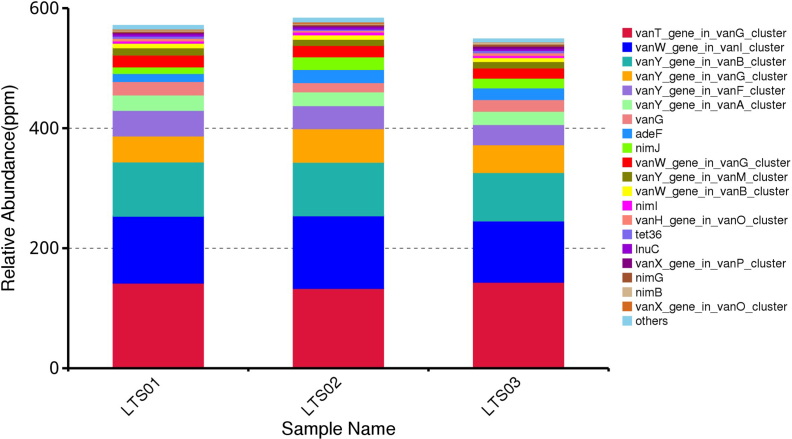
Relative abundance of ARGs in each sample, and the unit parts per million (ppm) is the result of amplification of the original relative abundance data by 106 times. Abbreviation: ARGs, antibiotic resistance genes.

**Fig. 10:**
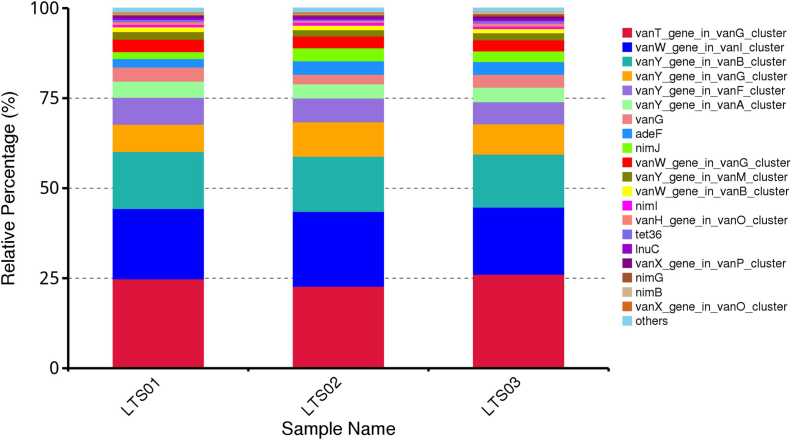
Relative abundance of the top 20 ARGs in all ARGs, and others represent the total relative abundance of non-top 20 ARGs. Abbreviation: ARGs, antibiotic resistance genes.

**Fig. 11:**
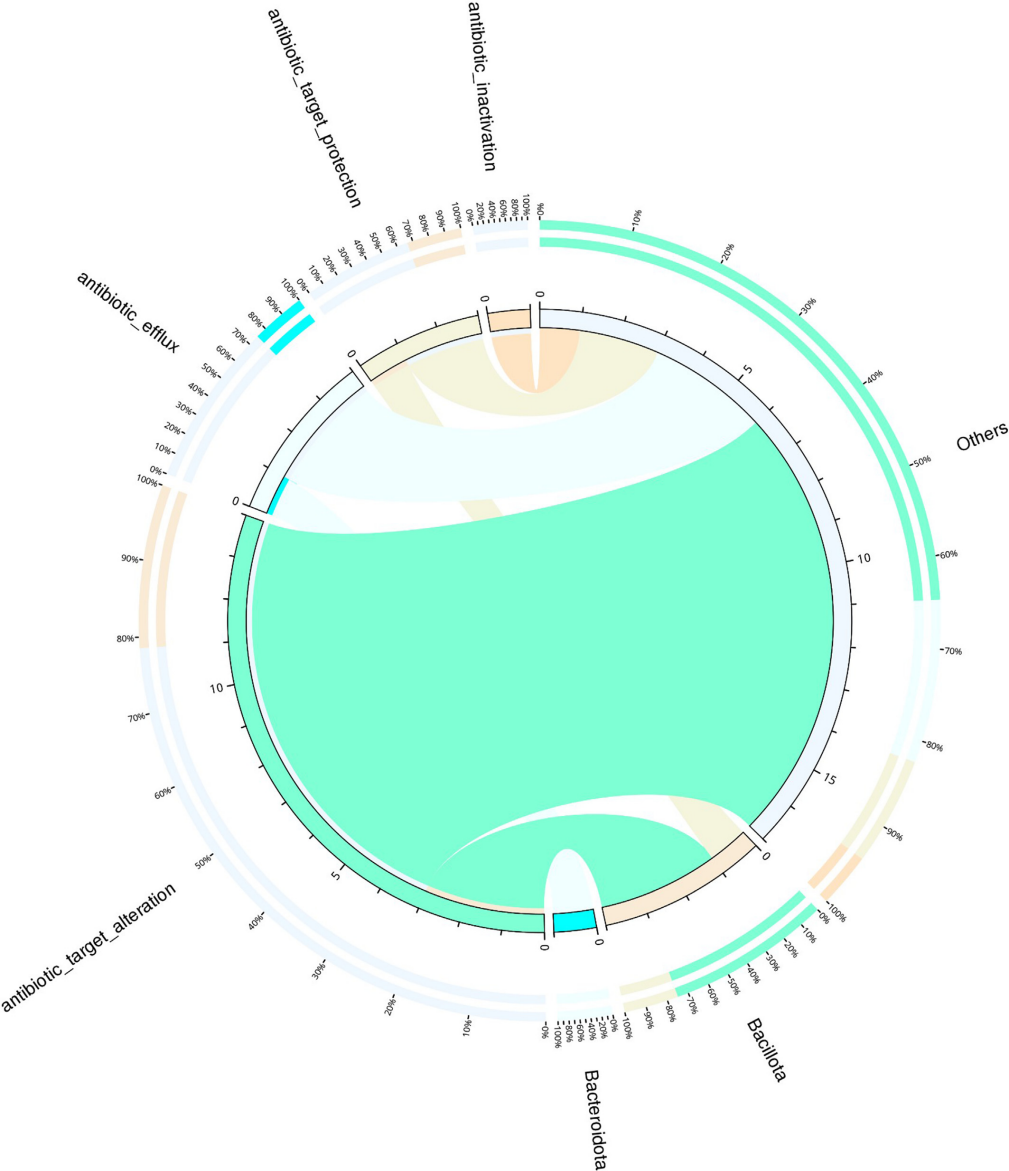
The circular diagram is partitioned into two distinct sections: the right side displays species information at the phylum level, while the left side presents resistance mechanism information. Within the inner circle, various colors are employed to represent different species and their corresponding resistance mechanisms. The scale utilized corresponds to the number of genes associated with each resistance mechanism. On the left-hand side, the sum of the number of resistance genes containing a specific resistance mechanism in each species is depicted, while the right-hand side showcases the cumulative number of resistance genes containing different resistance mechanisms within a particular species. Moving to the outer circle, the left side illustrates the relative proportion of resistance genes within each species with respect to their resistance mechanisms, providing an insight into the distribution of resistance mechanisms within individual species. Conversely, the right side depicts the relative proportion of resistance genes within each resistance mechanism, thereby revealing the prevalence of specific resistance mechanisms across different species.

Based on the analysis results using CARD annotation, we conducted an investigation into the species associated with drug resistance genes and identified the dominant bacterial flora carrying these genes. The comparison of bacterial gene sets and ARGs at the phylum level revealed 10% vs 15 %, 7% vs 2% for *Bacillota* and *Bacteroidota* respectively in LTS01; 6% vs 14%, 5% vs 2% for *Bacillota* and *Bacteroidota* respectively in LTS02; 6% vs 14% and 6% vs 2% for *Bacillota* and *Bacteroidota* respectively in LTS03 (Fig. 6). To visualize the proportion of antibiotic resistance ontology abundance in each sample and display the overall distribution, a concise overview circle diagram was generated (Fig. 7). Our clustered heat map results showed that the different samples were not clustered together (Fig. 8). Based on the abundance table of resistance genes, we determined the content and percentage of antibiotic resistance ontologies in each sample, and screened out the top 20 antibiotic resistance ontologies with the highest abundance as shown in Fig. 9, Fig. 10. Among the selected top 20 antibiotic resistance ontologies, the relative percentage and abundance of vancomycin resistance genes (*vanT* gene in *vanG* cluster, *vanW* gene in *vanI* cluster, *vanY* gene in *vanB*, *vanG*, *vanF*, *vanA*, and *vanM* cluster, *vanW* gene in *vanG* and *vanB* cluster, *vanH* gene in *vanO* cluster, *vanX* gene in *vanP* cluster, *vanX* gene in *vanO* cluster) were found to be highest in all samples followed by nitroimidazole resistance gene (*nimJ*, *nimI*, *nimG*, and *nimB* genes), multidrug resistance gene (*adeF*), and lincosamide resistance gene (*lnuC*) in all three fecal samples of the camels. Further, in order to understand the resistance mechanism by analyzing the relationship between the mechanism of action of the resistance genes and the corresponding species, a distribution map of the potential resistance mechanism was created using the CARD database classification (Fig. 11). Our results indicated that three major resistance mechanisms, i.e., antibiotic target alteration, antibiotic target protection, and antibiotic efflux, were associated with ARGs.

## 4 Discussion

Understanding the camel microbiome communities is important for enhancing camel health and productivity, as it reveals the microorganisms that aid in digestion and disease resistance. This knowledge can also lead to improved camel husbandry practices and nutritional strategies [30]. From the current study, the taxonomic analysis revealed that the camel fecal microbiome was predominantly bacterial (14%–20%), followed by viruses, archaea, and eukaryotes in descending order of abundance. This finding is consistent with other studies, demonstrating that bacterial species generally dominate the gut microbiota in mammals [31]. *Bacillota* and *Bacteriodota* were found to be the most abundant bacterial phyla, which are often observed to be abundant in gut microbiomes and play important roles in immune homeostasis and autoimmunity [32]. Members of the phylum *Bacillota*, formerly known as *Firmicutes*, produce enzymes that aid in the breakdown of complex carbohydrates and proteins. This phylum is particularly important in the fermentation of fibrous feed components common in the camel's arid environment [33] and contributes to the production of short-chain fatty acids (SCFAs), such as acetate, propionate, and butyrate, which are important sources of energy for the host [34]. Similarly, the phylum *Bacteroidota*, previously classified as *Bacteroidetes*, is also involved in the degradation of complex carbohydrates, proteins, and lipids. They have an extensive repertoire of carbohydrate-active enzymes (CAZymes), enabling them to efficiently process a broad range of dietary polysaccharides [35] as indicated by the unigene functional annotation results from the KEGG, EggNOG, and CAZy databases that highlight the diverse metabolic capabilities of the gut microbiota in the camel fecal samples.

The overrepresented metabolic pathways and other functional categories indicate a broad capacity for carbohydrate, amino acid, and lipid metabolism, as well as energy production. This aligns with the role of the gut microbiota in assisting the host in the breakdown and utilization of dietary compounds. For example, the presence of several abundant genera such as *Bacteroides*, *Ruminococcus*, and *Clostridium*, is noteworthy as they are crucial for fermenting indigestible dietary residues and producing SCFAs, which are key energy sources for the host [36]. *Bacteroides* and *Prevotella* are involved in the breakdown of proteins and carbohydrates, producing SCFAs [37]. *Ruminococcus* is involved in cellulose breakdown [36]. *Arthrobacter* and *Eubacterium* also contribute to the breakdown of complex carbohydrates and proteins [38]. Similarly, *Faecalibacterium* is a major butyrate producer, providing energy for the gut epithelium [39]. *Clostridium* species are involved in the degradation of dietary fiber, production of SCFAs, and fermentation [36]. *Alistipes*, *Oscillibacter*, and *Pseudoflavonifractor* have been associated with the metabolism of bile acids and fatty acids [40]. The high abundance of CAZymes, particularly glycoside hydrolases and glycosyl transferases, is consistent with the need for the degradation of complex carbohydrates found in the camel diet [26]. The enrichment in categories related to replication, recombination, repair, and translation underpins the genetic adaptability and replication capacity of the gut microbiota [41]. The overrepresentation of genes associated with inorganic ion transport and metabolism indicates the role of the microbiota in mineral absorption, while the enrichment of genes related to cell wall/membrane/envelope biogenesis and defense mechanisms suggested a role in maintaining gut integrity and host immune function [42] as indicated by the presence of *Akkermansia* genus which is involved in the regulation of gut barrier function and immune response [43]. Similar profiles of functional categories have been observed in the gut microbiomes of other herbivores such as cows and sheep [44]. Thus, these bacteria work synergistically to process a variety of dietary components, thereby contributing to the health and nutrient acquisition of their host. Overall, the composition and function of the camel fecal microbiome are well-adapted to the arid desert environment, assisting the camel in efficiently obtaining nutrients from its fibrous diet and contributing to its overall health and well-being.

The ARGs in the fecal samples were found to be predominantly from the bacterial phyla *Bacillota* and *Bacteroidota*. The finding of antibiotic resistance in these two phyla is consistent with other studies, as they are two of the most dominant phyla in mammalian gut microbiota. It has been suggested that these bacteria can horizontally transfer resistance genes, contributing to the prevalence of ARGs [45]. The increased occurrence of vancomycin resistance is of particular concern due to the antibiotic's role in treating severe infections. It's been reported that vancomycin-resistant bacteria can arise from the improper use of antibiotics, both in medical and agricultural practices [46]. Overuse or misuse of antibiotics in livestock can lead to antibiotic resistance in bacteria in their gut, which can spread to the environment via feces and possibly transfer to humans through various pathways [47]. In the study of the prevalence and dissemination of ARGs, tetracycline resistance genes were found to be abundant [48]. The differences between these studies could be attributed to several factors including regional variations in antibiotic use practices, different environmental conditions, and the methodologies used in the studies. For instance, regions with high antibiotic use in livestock farming might have a higher prevalence of ARGs in the environment, which can then be ingested by other animals like camels [49]. Resistance mechanisms identified in the study, such as antibiotic target alteration, antibiotic target protection, and antibiotic efflux, align with known mechanisms. These strategies used by bacteria often involve altering the target site of the antibiotic, reducing the intracellular concentration of the antibiotic, or modifying the antibiotic molecule itself [50].

The potential for environmental contamination by antibiotic-resistant bacteria is a growing concern due to widespread antibiotic misuse and the shedding of resistant bacteria and ARGs into the environment. In the current study, the fecal microbiome of camels was found to harbor a significant amount of ARGs, particularly those associated with resistance to vancomycin, nitroimidazole, multidrug resistance, and lincosamides. These findings align with other studies in other livestock and wildlife species that have detected high levels of ARGs in fecal samples. While the gut microbiome is a natural reservoir for ARGs, the use of antibiotics can select for resistant strains, potentially enriching the abundance of these genes in the microbiome. Once these ARGs enter the environment, they can potentially spread to other bacteria through horizontal gene transfer (HGT), including bacteria that are pathogenic to humans. HGT is thought to play a crucial role in the spread of antibiotic resistance in the environment [51]. Fecal contamination of the environment, particularly water sources, can introduce ARGs into new ecosystems. This can lead to the dissemination of ARGs into microbial communities that had previously been unexposed to these genes. A study had found a significant amount of ARGs in fecal-contaminated river water, demonstrating the potential for environmental contamination [52]. The detection of ARGs, including vancomycin resistance genes, in camel fecal microbiome could have implications for environmental health, particularly in regions where camel rearing is prevalent. Overuse and misuse of antibiotics in veterinary medicine could exacerbate this issue by selecting for resistant strains in livestock microbiomes, which can then be shed into the environment. Therefore, it is essential to monitor and regulate the use of antibiotics in livestock to mitigate the spread of antibiotic resistance. Research like this study that reveals the extent of antibiotic resistance in different ecosystems is a crucial step towards understanding and controlling this global issue.

## Conclusion

In conclusion, this study offers important insights into the camel fecal microbiome, emphasizing its adaptability to arid desert environments and its role in aiding nutrient extraction from a fibrous diet. The prevalence of bacterial species, particularly within the phyla *Bacillota* and *Bacteroidota*, highlights their essential function in breaking down complex carbohydrates and proteins and producing SCFAs, which are a key energy source for camels. Additionally, the study reveals the presence of ARGs in the camel fecal microbiome, with *Bacillota* and *Bacteroidota* serving as the main reservoirs. The detection of ARGs raises concerns about environmental contamination, especially in regions where camels are commonly raised. The fecal shedding of antibiotic-resistant bacteria and ARGs into the environment can facilitate the spread of resistance to other bacterial populations, including human pathogens. Therefore, it is critical to monitor and regulate antibiotic use in livestock to prevent the proliferation and dissemination of antibiotic-resistant strains.

## Data availability

The corresponding author can be contacted to request the data by submitting a reasonable inquiry.

## CRediT authorship contribution statement

**Yan Gao:** Conceptualization, Data curation, Formal analysis, Funding acquisition, Investigation, Methodology, Project administration, Resources, Software, Supervision, Validation, Visualization, Writing – original draft, Writing – review & editing. **Jiangchao Wu:** Conceptualization, Data curation, Funding acquisition, Investigation, Resources, Software, Visualization, Writing – original draft, Writing – review & editing. **Jun Zeng:** Conceptualization, Data curation, Funding acquisition, Investigation, Methodology, Project administration, Resources. **Xiangdong Huo:** Conceptualization, Data curation, Formal analysis, Methodology, Project administration, Resources, Supervision, Validation, Visualization, Writing – original draft, Writing – review & editing. **Kai Lou:** Conceptualization, Data curation, Formal analysis, Funding acquisition, Investigation, Methodology, Project administration, Resources, Software, Supervision, Validation, Visualization, Writing – original draft, Writing – review & editing.

## Declaration of competing interest

The researchers affirm that there are no conflicts of interest associated with this study.
