# Supplementary material for: Beyond the desert sands: decoding the relationship between camels, gut microbiota, and antibiotic resistance through metagenomics
Source: Sci One Health. 2024 Jul 1;3:100071. doi: 10.1016/j.soh.2024.100071 (PMC11701853; doi:10.1016/j.soh.2024.100071)
Supplement: Multimedia component 1 [file mmc1.docx]

Figure S1: Each sample has been individually analyzed, with the calculated scaftig lengths presented in a plotted graph. The vertical axis demonstrates the frequency (in count), while the horizontal axis represents the length of the scaftigs. A yellow curve is used to depict the total number and corresponding percentage of scaftigs.


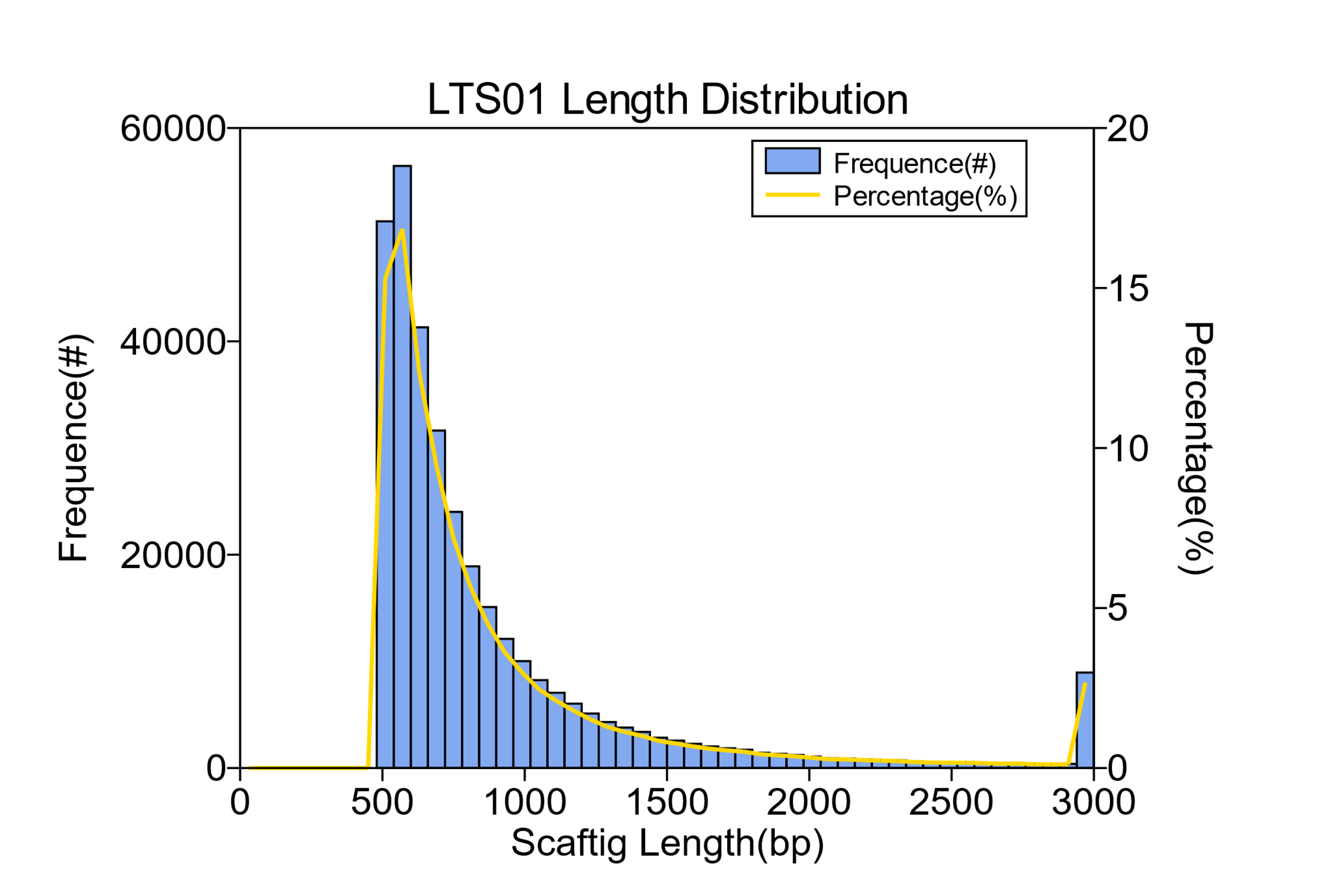

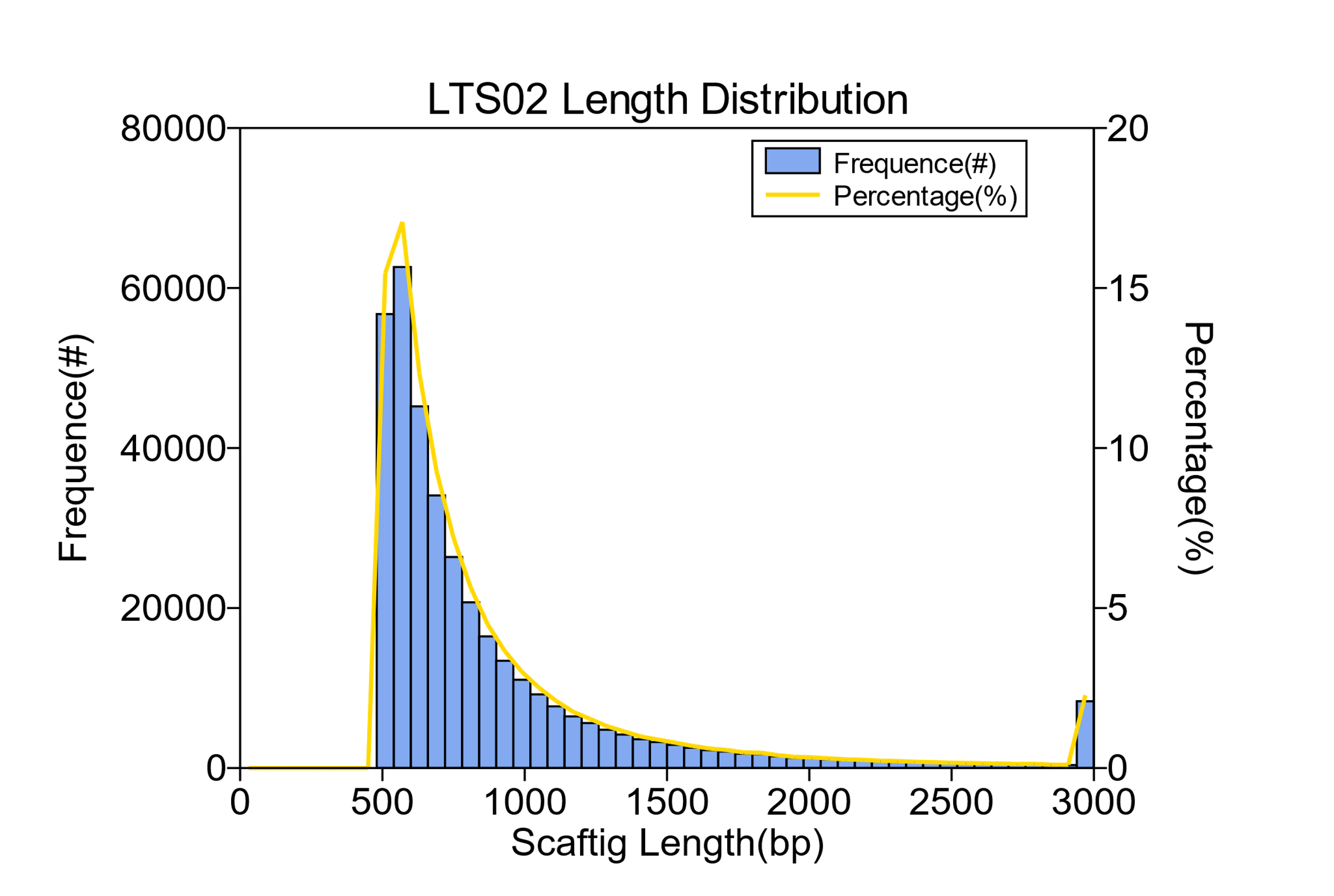

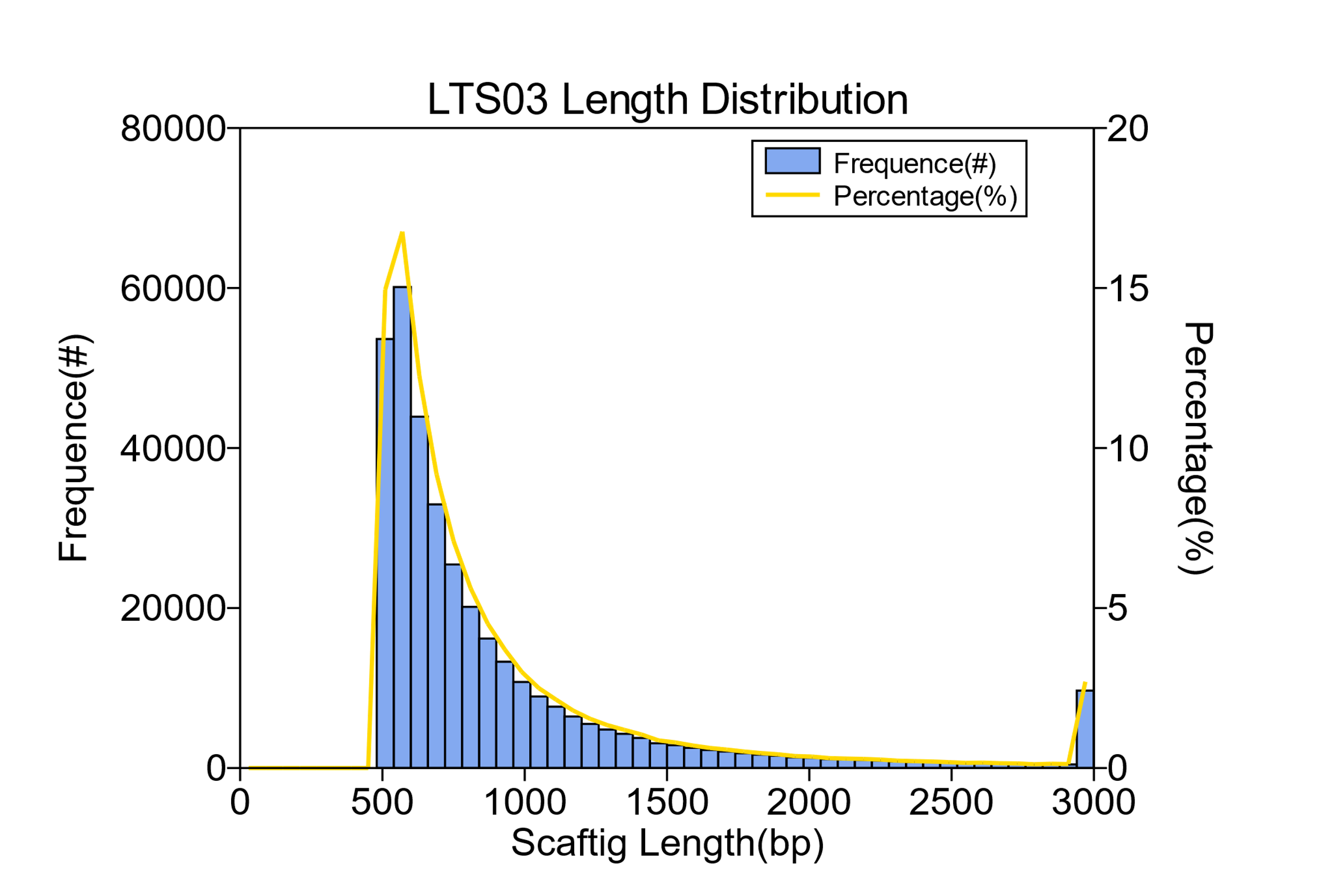


Figure S2: Each sample has been individually analyzed, with the calculated gene lengths presented in a plotted graph. The vertical axis demonstrates the frequency (in count), while the horizontal axis represents the length of the genes. A yellow curve is used to depict the total number and corresponding percentage of genes.


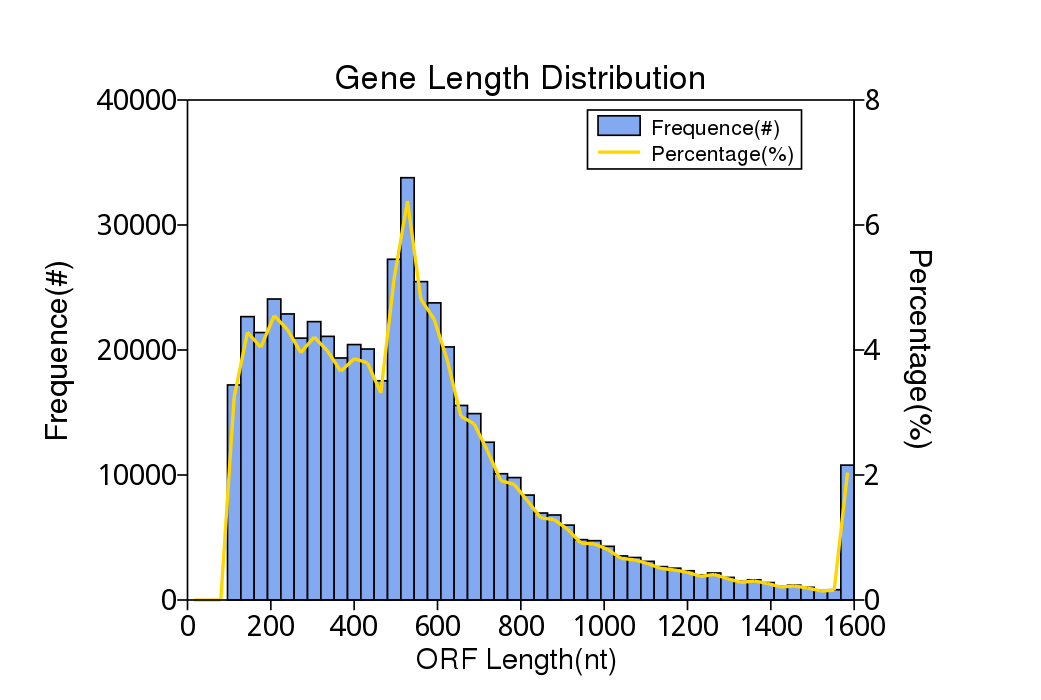


Sample LTS01


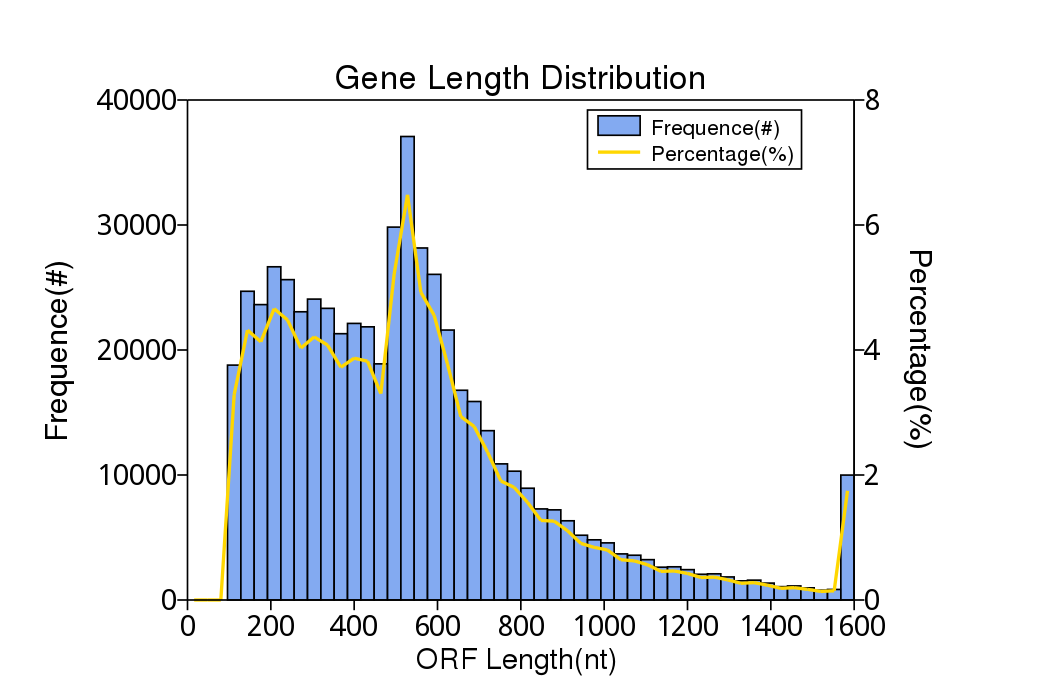


Sample LTS02


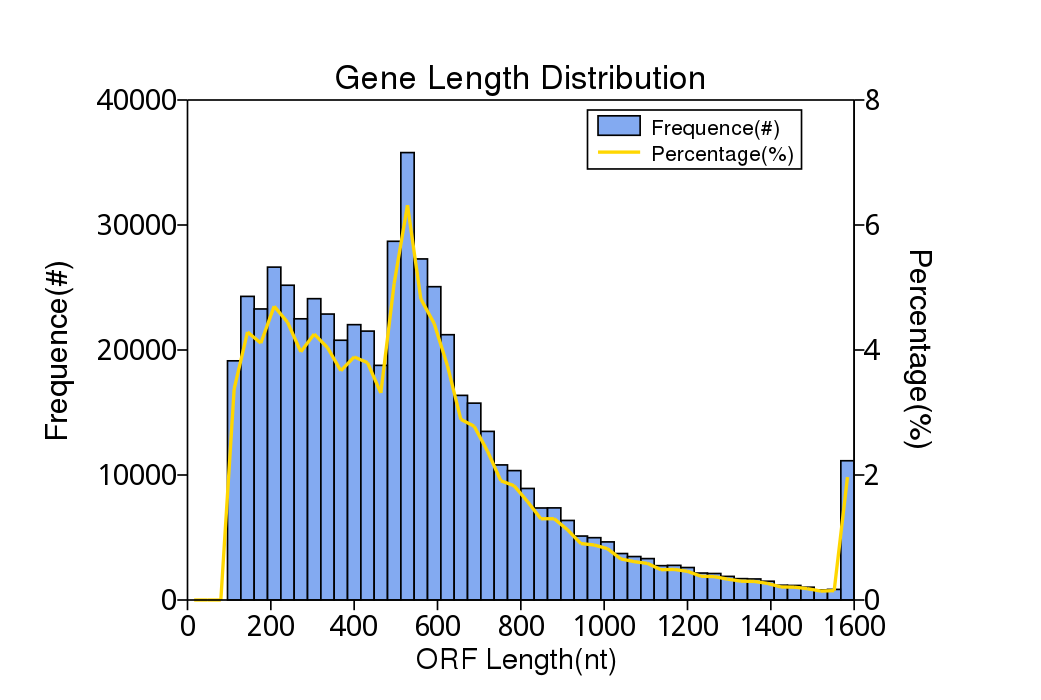


Sample LTS03


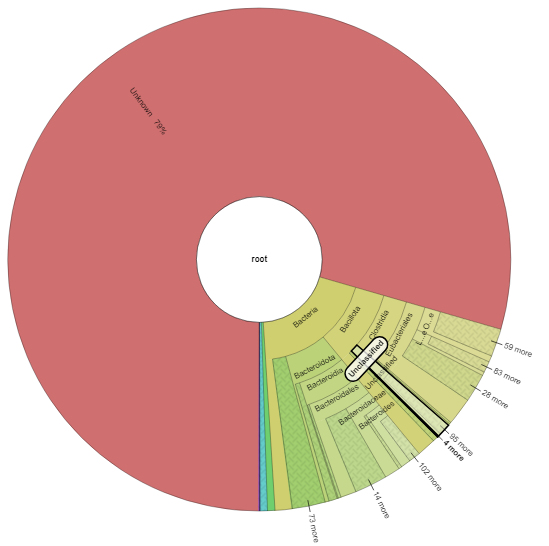


Sample LTS01


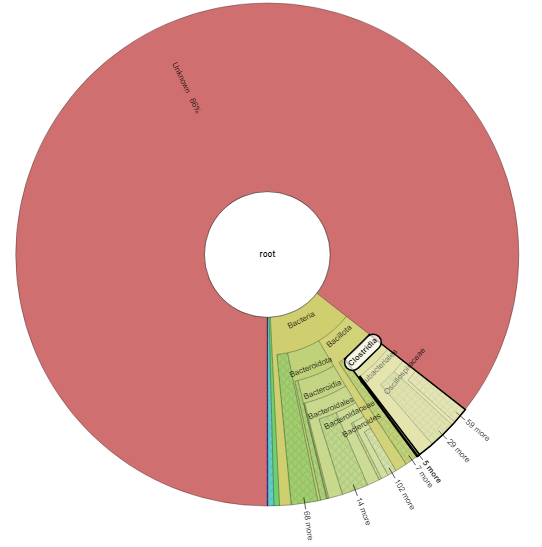


Sample LTS03


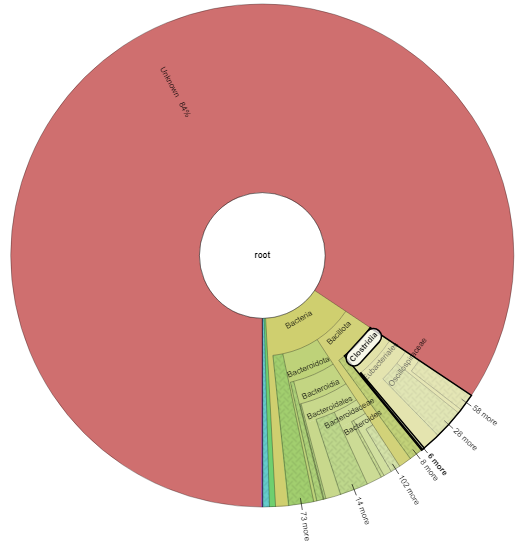


Sample LTS02

Figure S3: The fecal microbiota composition of camels dominated by bacteria followed by viruses, archaea, and eukaryota


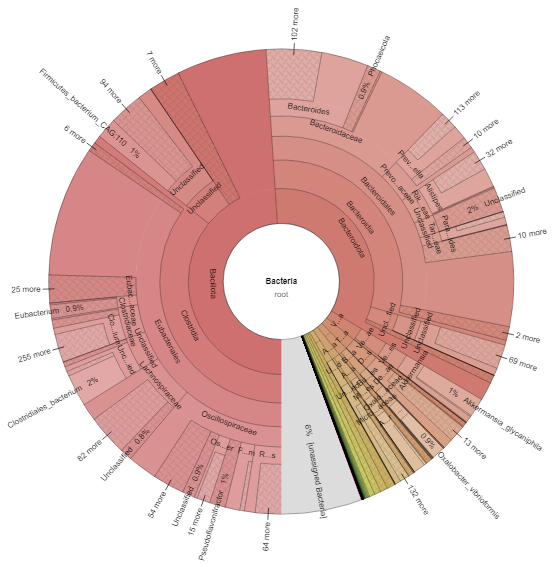

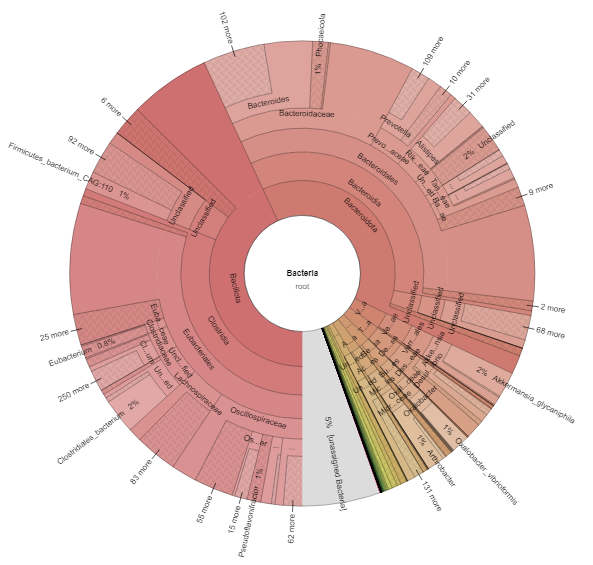

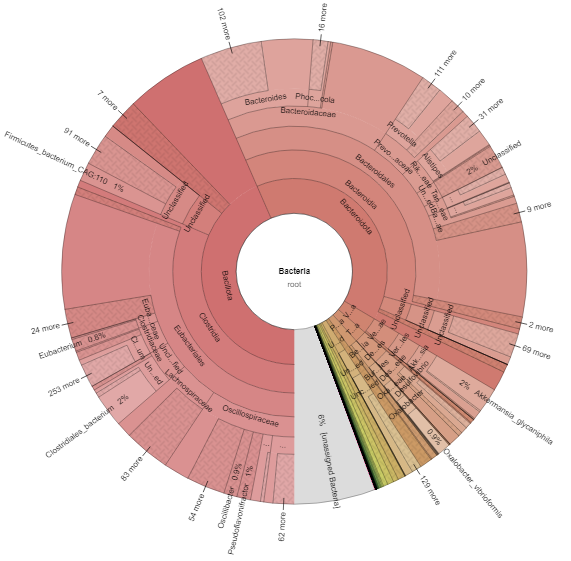


Sample LTS01

Sample LTS02

Sample LTS03

Figure S4: The fecal microbiota composition of camels. The most abundant phyla were Bacillota and Bacteriodota


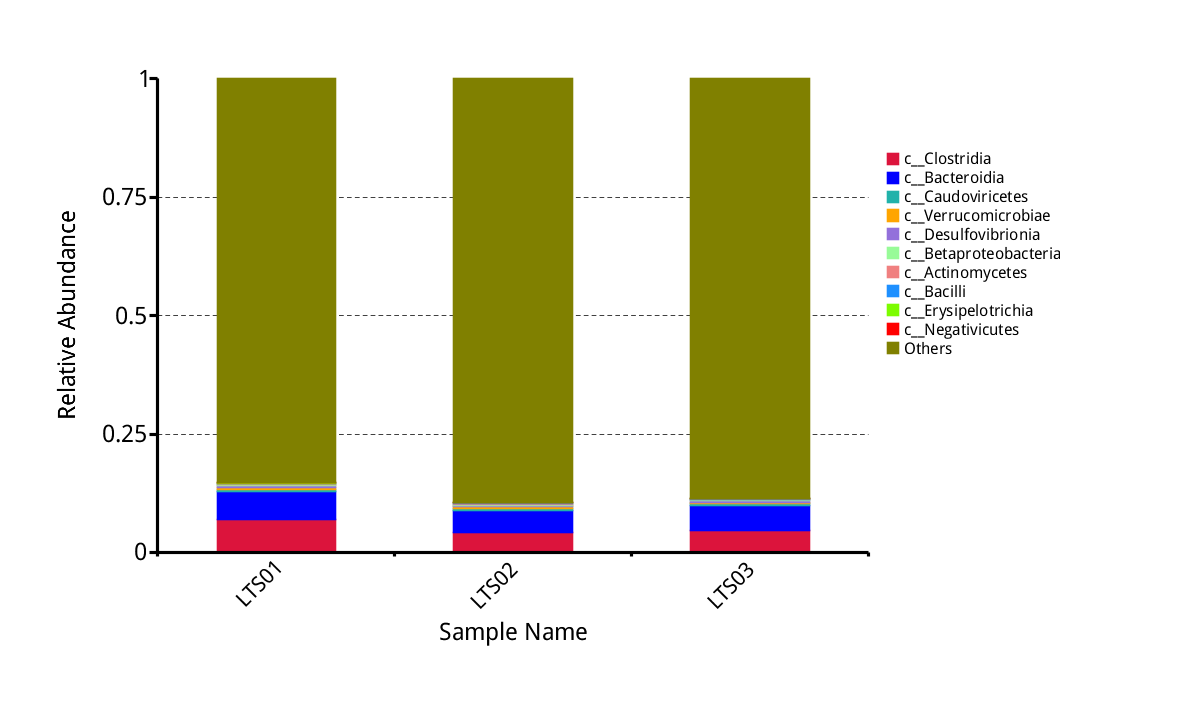

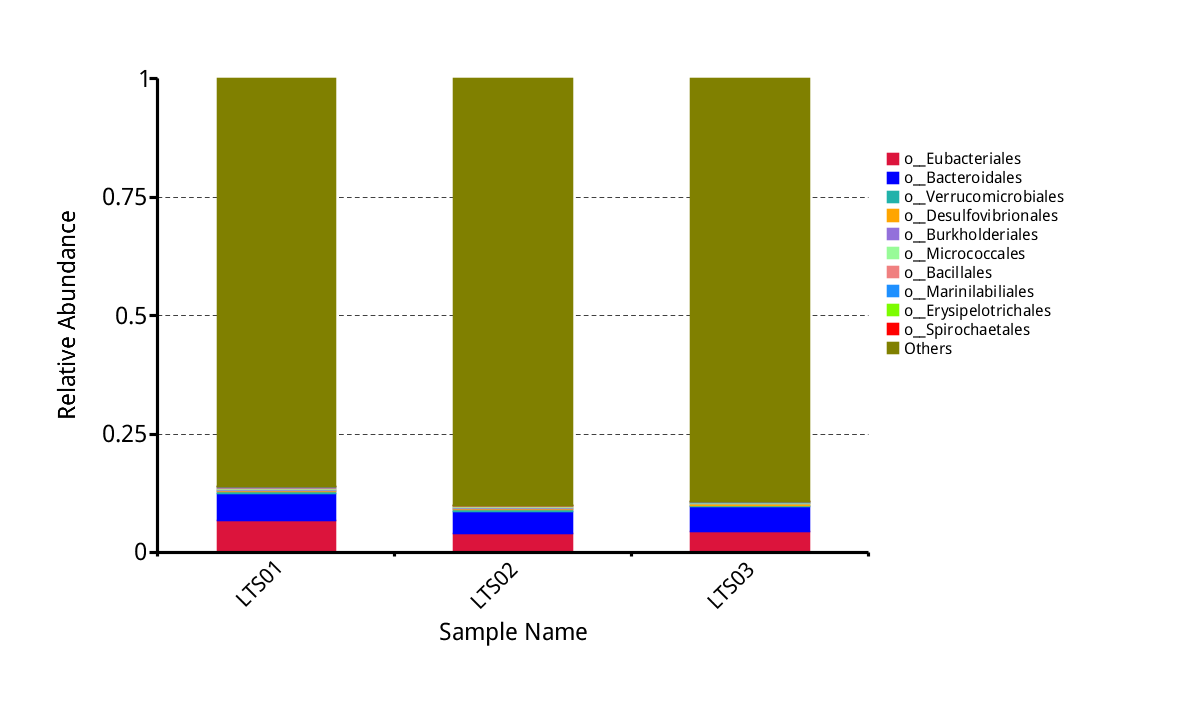

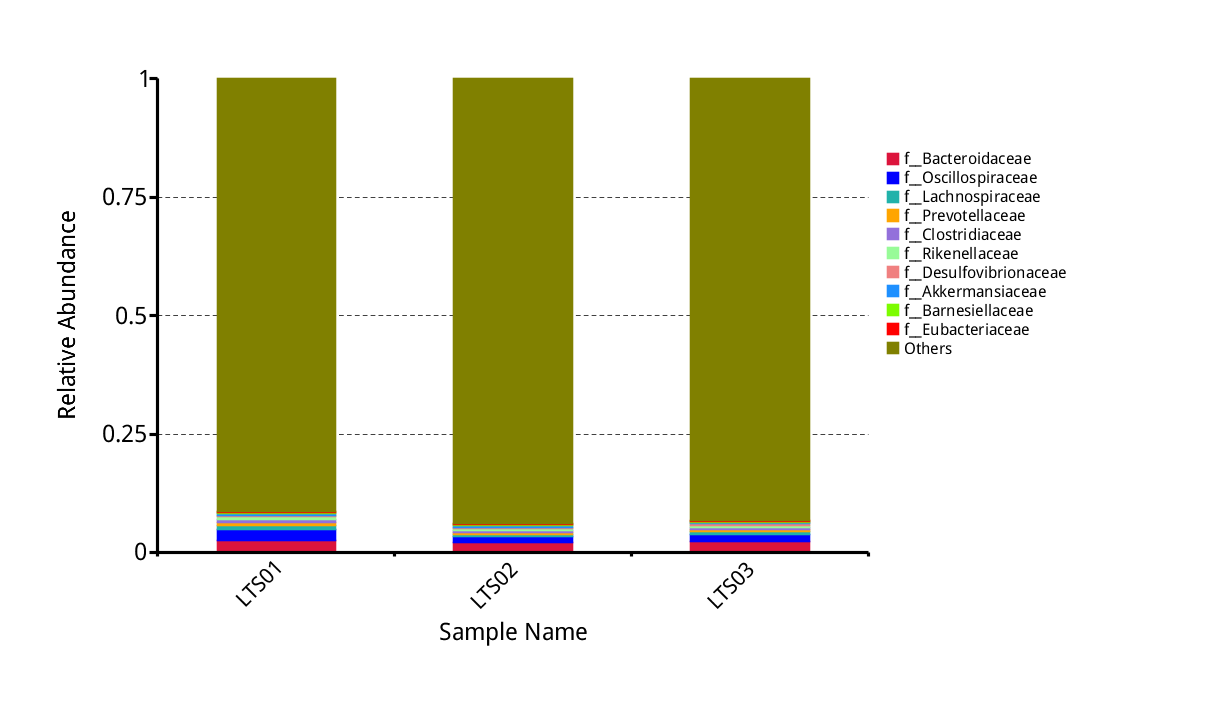

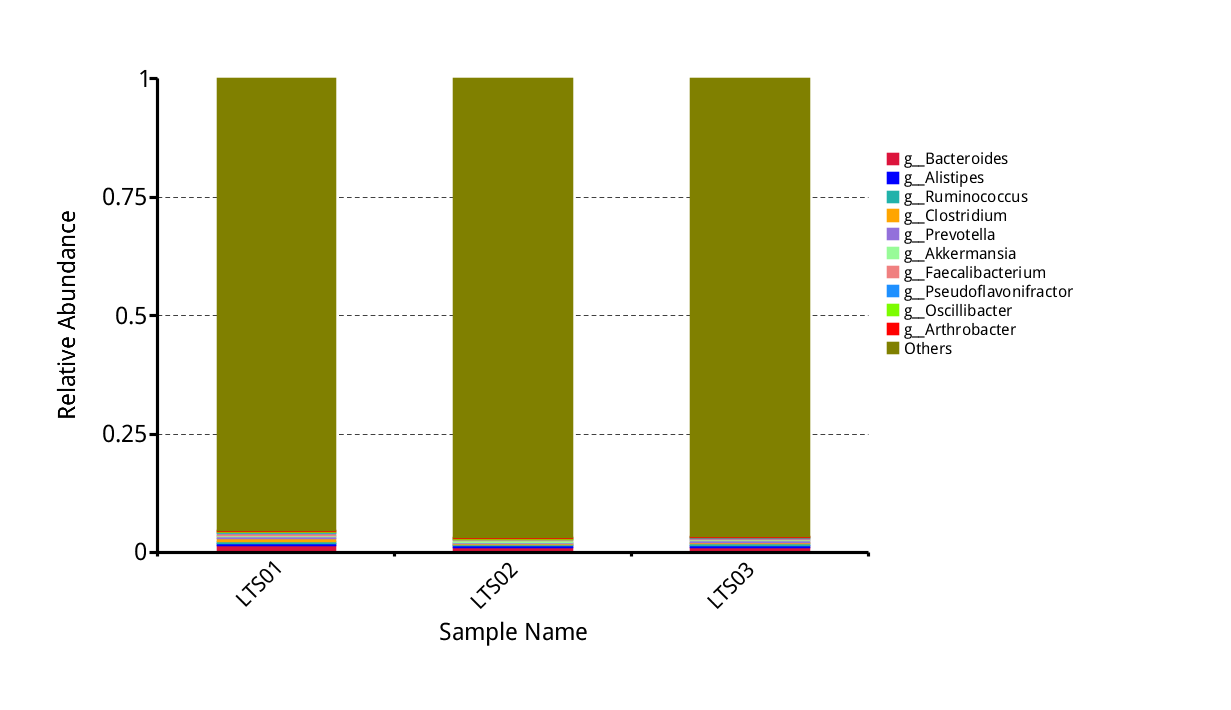

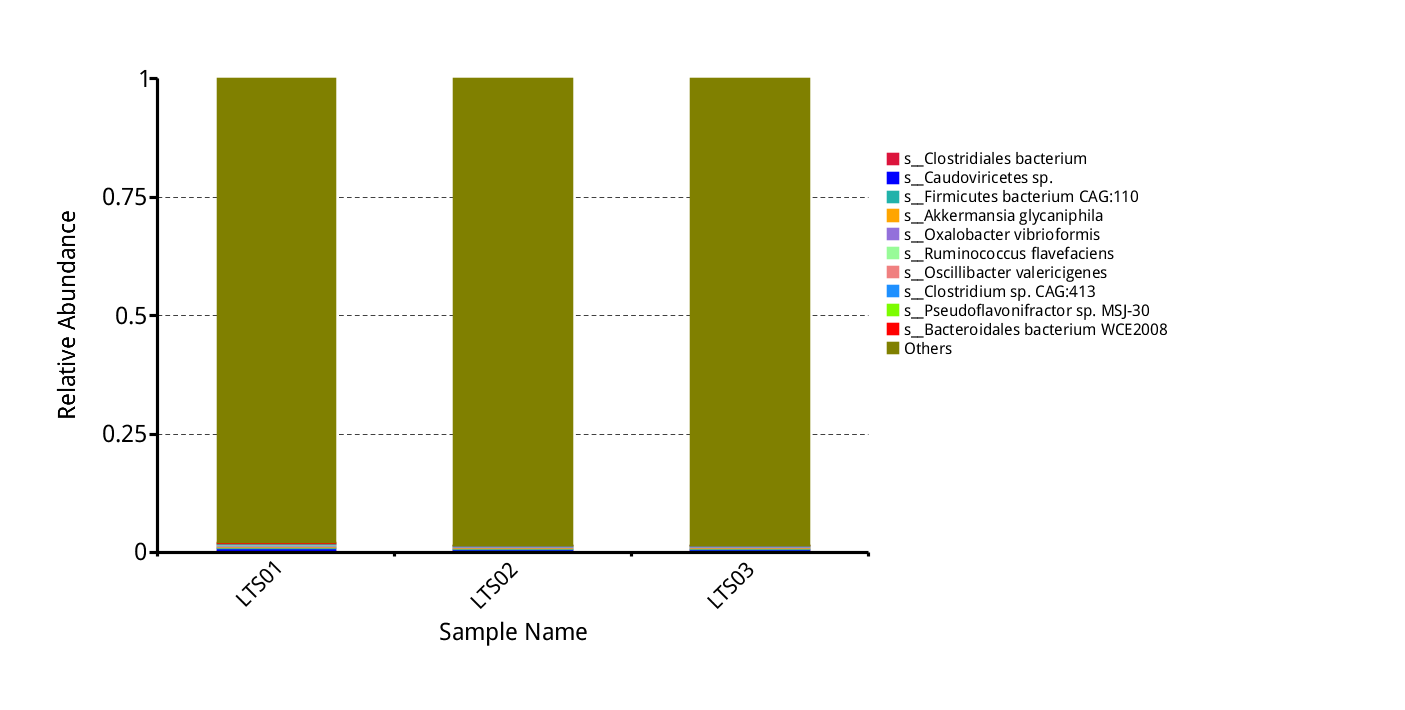


A

E

D

C

B

Figure S5: Relative abundance of fecal microbiota of camel at different taxonomic levels: (A) Class, (B) Order, (C) Family, (D) Genus, (E) species in three samples.

Table S1: raw and clean base data

| Sample ID | Insert Size (bp) | Raw Data | Clean Data | Clean_Q20 | Clean_Q30 | Clean GC (%) | Effective (%) |
| --- | --- | --- | --- | --- | --- | --- | --- |
| LTS01 | 350 | 6,211.45 | 6,196.99 | 97.6 | 93.22 | 46.27 | 99.767 |
| LTS02 | 350 | 6,489.16 | 6,466.99 | 97.61 | 93.28 | 46.88 | 99.658 |
| LTS03 | 350 | 6,600.23 | 6,573.91 | 97.6 | 93.23 | 45.77 | 99.601 |

Table S2: scaftigs information

| Sample ID | Total length (bp) | Number | Average length (bp) | N50 Length (bp) | N90 Length (bp) | Max length (bp) |
| --- | --- | --- | --- | --- | --- | --- |
| LTS01 | 324,923,946 | 335,012 | 969.89 | 951 | 550 | 130,864 |
| LTS02 | 344,209,851 | 366,988 | 937.93 | 920 | 547 | 123,566 |
| LTS03 | 345,394,683 | 358,644 | 963.06 | 953 | 550 | 130,668 |

Table S3: Information of open reading frame

| Sample ID | ORFs NO | without stop-and-start codons | Stop codons | Start codons | Both start and stop codons | Total Length (Mbp) | Average Length (bp) | GC % |
| --- | --- | --- | --- | --- | --- | --- | --- | --- |
| LTS01 | 529,190 | 77,689(14.68%) | 135,652(25.63%) | 184,954(34.95%) | 130,895(24.73%) | 289.14 | 546.39 | 47.8 |
| LTS02 | 571,971 | 85,405(14.93%) | 148,522(25.97%) | 202,538(35.41%) | 135,506(23.69%) | 306.32 | 535.55 | 48.14 |
| LTS03 | 566,527 | 81,342(14.36%) | 146,622(25.88%) | 199,336(35.19%) | 139,227(24.58%) | 306.91 | 541.74 | 46.85 |
